# Resection Leads to Less Recurrence Than Strictureplasty in a Paediatric Population with Obstructive Crohn's Disease

**DOI:** 10.1155/2014/709045

**Published:** 2014-04-01

**Authors:** Richard Bamford, Ashley Hay, Devinder Kumar

**Affiliations:** Department of General and Colorectal Surgery, St George's Healthcare NHS Trust, Blackshaw Road, Tooting, London SW17 0QT, UK

## Abstract

*Introduction*. Resection and strictureplasty are used to treat patients with obstructive Crohn's disease. Strictureplasty is preferable in adults as it retains bowel length. This study aims to identify differences in outcomes of children undergoing strictureplasty and resection for obstructive Crohn's disease. *Method.* Patients under 20 years undergoing surgery over a nine-year period were included. Data was collected on procedures for stenotic Crohn's disease. Patients were divided into 2 groups: Group 1 treated with strictureplasties and Group 2 resections. Postoperative complications and recurrence rates were recorded. Kaplan-Meier method was used to analyze the data. *Results*. Twenty-six patients and 40 operations were identified. Mean age was 15.6 years (7.2–19.4) with equal numbers of males and females. Mean follow-up was 45.9 months (0.1–149.9). 20/40 procedures involved the terminal ileum; 9/40, the ileocolic junction; 8/40, the upper GI tract; and 3/40, the colon. Group 1 consisted of 19 strictureplasties and Group 2 consisted of 13 resections and 8 combined procedures. Significantly more patients in Group 1 required further surgery (11/19 versus 3/21; *P* = 0.008). *Conclusion*. Allowing for variations in disease duration, severity, and previous medical management, these data suggest that resection is preferable to strictureplasty in treating obstructive Crohn's disease in children and adolescents.

## 1. Introduction

Children and adolescents account for 25% of all patients with Crohn's disease and can have a significant impact on growth and development [1]. Over their lifetime, affected patients have a 70–90% chance of surgical intervention [2, 3]. Furthermore, recrudescence of the disease requiring additional surgical intervention can occur in 50% of patients [4, 5].

Indications for surgical intervention in Crohn's disease include perforation, abscess formation, bleeding, malignancy, and fibrotic strictures [6]. This study aims to concentrate on the outcomes of patients treated for the latter of these indications. Strictureplasty and resection are both used to treat obstructive Crohn's disease.

First described in the 1970s as a treatment for tuberculosis, strictureplasty was employed for the management of Crohn's strictures in the early 1980s [7, 8]. The indications for its use are to preserve small bowel length in patients who would otherwise require a large resection, single site fibrotic strictures in inactive disease, recurrence of strictures less than 1 year since resection, isolated ileocolonic strictures, and selected duodenal strictures [9, 10]. The most commonly performed strictureplasty is the Heineke-Mikulicz procedure that is most suitable for strictures less than 10 cm although other techniques have been successful in lengths up to 90 cm [11, 12]. In an adult population, its use has been proven to be both safe and effective [13, 14]. The main benefit is to retain bowel length [15]. Because of this risk, resection is not considered the optimal surgical technique. However, it has been suggested that resection may be beneficial in adults with early isolated disease [16, 17]. An alternative treatment option is endoscopic dilatation of the stricture. This may be useful with short segments (<4 cm) but its use is limited with mixed results that have not yet been shown to be of benefit [18, 19].

There is little evidence that suggests the most appropriate surgical technique for obstructive disease in children or the risk factors that may precipitate the need for further surgical intervention. This study therefore aims to identify the outcomes in children undergoing strictureplasty and resection for obstructive Crohn's disease and to identify areas that may improve surgical outcomes.

## 2. Method

A retrospective review of case notes and electronic records of consecutive patients undergoing colorectal surgery at a London teaching hospital over a period of nine years was performed. The inclusion criteria for this study were patients under 20 and having undergone surgery for stenotic Crohn's disease during the study period. Recurrence or recrudescence of the disease was defined as the need for further surgical intervention after failed medical therapy.

Follow-up was not predetermined but routinely involved a 3-month outpatient appointment with the surgical team who performed the surgery and subsequent 6-month to 1 year appointments thereafter with either the surgical or medical teams involved in the patients care. If required, the patient's general practitioners could arrange more urgent appointments.

A single member of the surgical team recorded all the information in an anonymized electronic database.

Preoperative information collected included patient demographics, previous procedures, and preoperative weight. Intraoperative findings included the number of strictures, intraoperative complications, and type of procedure performed. Postoperative data was recorded from hospital stay, routine follow-up, and subsequent emergency admissions. Postoperative complications and recurrence rates were recorded.

Patients were then divided into two groups identifying patients treated with strictureplasty alone as Group 1 and patients undergoing resection as Group 2.

### 2.1. Statistical Analysis

The two groups were analyzed using the Kaplan-Meier method with Mantel Cox methods employed to compare the two groups' episodes of recurrence.

## 3. Results

Twenty-six consecutive patients were identified for inclusion and no patients were excluded. The mean age of patients was 15.6 years (7.2–19.4) with an equal number of males and females. Mean follow-up time was 67.4 months (10.5–156.6).

Of these patients, 8/26 required additional surgical interventions resulting in 40 procedures being performed. All surgical interventions were performed by the same consultant surgeons or under their direct supervision. 5/8 patients required one further surgical intervention and three other patients required two, three, and four subsequent procedures, respectively. In total, 23/40 procedures were in female patients and 17/40 were in male patients.

The overall recurrence or recrudescence rate was 35% (14/40). Mean time to recurrence or recrudescence was 29.1 months (2.6–75.0). 9/14 patients had recurrence at a different site to the original and 5/14 had recrudescence at the same site. The recurrence rate in females was 43% (10/23) and 23.5% (4/17) in males. This was not found to be statistically significant (*P* = 0.201).

Half of the (20/40) procedures involved the terminal ileum, 9/40 the ileocolic junction, 8/40 the upper gastrointestinal tract, and 3/40 the colon. Mean length of stricture was 12.4 cm (5–30 cm). Site of the initial disease had no significant impact on recurrence of disease (*P* = 0.541). The most common site resulting in recrudescence was the terminal ileum group (8/20), and no recurrences occurred in the colon group. Ileocolic junction recurrences occurred in 3/9 and the upper gastrointestinal tract occurred in 3/8 procedures.

Strictureplasties alone accounted for 19/40 procedures and made up Group 1. Seven were Finney procedures and 12/19 were Heineke-Mikulicz procedures. Five of each required reintervention (*P* = 0.742). 13/40 resections were performed, and 8/40 were combined procedures with both sets of procedures making up Group 2. There were no mortalities in either group and surgical complications are identified in Table 1.

**Figure 1:**
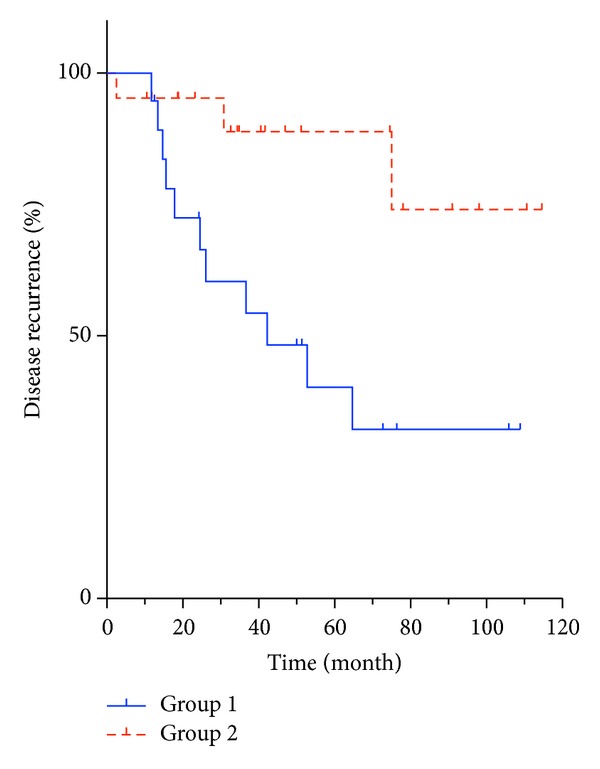
Kaplan-Meier curve for disease recurrence needing further surgical intervention. Significantly fewer patients who had resections (Group 2) required further intervention (*P* = 0.008).

The overall rate of recrudescence requiring surgical intervention was 35% (14/40). 11/14 were from Group 1 and 3/14 were from Group 2. In Group 1, 11/19 cases required further surgical intervention, which is significantly greater than the 3/21 cases in Group 2 (*P* = 0.008, Figure 1). The characteristics of patients in each group are presented in Table 2.

## 4. Discussion

Crohn's disease is an incurable inflammatory disease that can affect any part of the digestive tract and which affects a large number of children and adolescents. Despite the improving medical therapy, nearly all of these patients will require surgery within 10 years of diagnosis [2, 3]. A majority of these are likely to require further surgical management [4, 5].

Surgical management for Crohn's disease includes both resection and strictureplasty for stenotic disease. In an adult population, strictureplasty has been consistently proven to be safe and effective [13, 14, 20, 21]. Recrudescence and complication rates are consistently shown to have no significant difference to that of resection even when active disease is present at the resection margin [14, 20, 22, 23]. Even in studies where strictureplasty shows shorter interval to reoperation [24], the main benefit is to retain bowel length. This is particularly important in patients at risk of short bowel syndrome and a major factor as to why strictureplasty is often considered the surgery of choice for stenotic Crohn's [15]. Despite this, resection has its place especially in areas of bowel where there is significant active disease. It has also been suggested that resection may be useful in adults with early isolated disease [16, 17]. If it is performed, then as small a section as possible should be removed and macroscopically active disease can be left behind without increasing the recrudescence rates [4, 25].

This study demonstrates a significant advantage of resection over strictureplasty in children with obstructive Crohn's disease in terms of reoperation free survival. A recent article by Barrena et al. supports this. Although limited by small numbers, they showed children who underwent surgery for Crohn's disease had improved nutritional status, increased growth, and improved quality of life postoperatively. The majority of their population was treated with resection [26]. The results in this study may be related to the retention of active disease in strictureplasty patients. Patients of a young age and with recent disease diagnosis are considered to be at high risk of early recrudescence of disease [20, 27, 28]. This may suggest an increase in disease activity in this population and therefore higher associated risk with retaining the diseased bowel.

Other risk factors are also reported in the literature. Smoking is often associated with an increase in disease recurrence [27, 28]. This was not assessed in this population due to their age range and lack of information from patient's records. We concede, however, that smoking cessation and prevention advice should be included in the general management of this young population. Gender is also a considered risk factor with females more likely to suffer complications than males [29]. This was not found to be significant in this study. Site of disease is also quoted as a risk factor [24] but it has not been found to be significant in this series.

Accurately assessing the recurrence rates for Crohn's is limited by variations in definitions and follow-up times in the literature. The overall recurrence rate in our study was 35%, which is consistent with other studies that have investigated an adult population [20, 21].

The risk of recurrence in the strictureplasty group was higher than the reported data. However, the resection group was consistent with the accepted recrudescence rates for strictureplasty in adults [14, 20]. In a population who commonly requires multiple operations and whose cumulative risk of surgery increases with age, this study would suggest that resection rather than strictureplasty may lead to improved outcomes and a reduced risk of further surgical intervention [29, 30].

A limitation to this study is the lack of information regarding the medical management of the population prior to and after surgical intervention. Benchimol et al. have recently suggested that optimizing medical management in children with Crohn's disease, especially those first diagnosed when they are older than 10 years, reduces the need for surgical intervention [31]. In this study population, experienced specialist paediatric gastroenterologists initially assessed all patients, and medical management followed the guidelines from the British Society of Gastroenterologists. This would involve immunosuppressant and disease modifying medications including adequate nutrition, corticosteroids, mesalazine, and infliximab which were appropriate. As all patients within the groups required further surgical intervention at the end of failed medical management, then any differences can be assumed to be minimal.

Surgical intervention remains an important aspect of Crohn's management in children. A combined approach involving both appropriate medical and surgical approaches would be recommended in Crohn's management in children.

## 5. Conclusion

Surgical intervention continues to be a vital component of the management of children with Crohn's disease. When combined with optimized medical management, resection of strictures is preferable to strictureplasty in treating obstructive Crohn's disease in children.

## Figures and Tables

**Table 1:** Complications of patients undergoing surgical intervention.

	Strictureplasty (*n* = 19)	Resection (*n* = 13)	Combined (*n* = 8)	Total (*n* = 40)
Abscess	2	0	0	2
Anastomotic leak	1	1	1	3
Dehiscence	0	0	0	0
Fistula	0	0	1	1
Back to theatre	1	0	1	2
Low respiratory tract infection	1	0	2	3
Urinary tract infection	0	0	0	0
Pulmonary embolism	0	0	0	0
Deep vein thrombosis	0	0	0	0
Ileus	1	1	0	2
Death	0	0	0	0

**Table 2:** Characteristics of all procedures, of Group 1, strictureplasty, and of Group 2, resection.

	Total (*n* = 40)	Group 1 (*n* = 19)	Group 2 (*n* = 21)
Gender (male : female)	17 : 24	8 : 11	9 : 12
Age (mean, years)	15.57 (7.2–19.4)	15.6	14.8
Time to follow up (months)	67.44 (10.5–156.6)	74.4	61.1
Number of strictures (mean)	3 (1–14)	4.6 (1–14)	1.5 (1–6)
Stricture length (mean, cm)	12.4 (5–30)	10.2 (5–30)	14.85 (5–30)
Stricture site	20/40 ileum 9/40 ileocolic junction 8/40 upper GI tract 3/40 colon	10/19 ileum 4/19 ileocolic junction 5/19 upper GI tract 0/19 colon	10/21 ileum 5/21 ileocolic junction 3/21 upper GI tract 3/21 colon
Recurrence	14/40	11/19	3/21
Time to recrudescence (months)	29.07 (2.6–75.0)	26.97 (11.8–64.7)	36.76 (2.6–75.0)
Same site recrudescence	5	3	2
Different site recrudescence	9	8	1
